# Quantitative gas-phase separation of carrier-free thallium radioisotopes from proton-irradiated ${^\textrm{nat}\textrm{HgO}}$

**DOI:** 10.1007/s10967-025-10125-y

**Published:** 2025-05-07

**Authors:** Jennifer M. Wilson, Dominik Herrmann, Pascal V. Grundler, Nicholas P. van der Meulen, Alexander Sommerhalder, Patrick Steinegger

**Affiliations:** 1 Laboratory of Inorganic Chemistry, Department of Chemistry and Applied Biosciences, ETH Zürich, Zürich, Switzerland; 2 PSI Center for Nuclear Engineering and Sciences, Paul Scherrer Institute, Villigen PSI, Switzerland; 3 PSI Center for Life Sciences, Paul Scherrer Institute, Villigen PSI, Switzerland

**Keywords:** Mercury oxide, Decomposition, Thallium, Radioisotope, Radionuclide, Production, Separation

## Abstract

Carrier-free ${^{202}\textrm{Tl}}$ was thermally separated from proton-irradiated ${^\textrm{nat}\textrm{HgO}}$. Pressed ${^\textrm{nat}\textrm{HgO}}$ targets were irradiated with $\approx {26}\,\textrm{MeV}$ protons at the IP2 beamline of the high-intensity proton accelerator facility at the Paul Scherrer Institute. The well-known thermal decomposition of $\textrm{HgO}$ at $>{470}\,^\circ$C and the strong adsorption of $\textrm{Tl}$ on a $\textrm{Ta}$ surface allowed for a simple and quantitative gas-phase separation of carrier-free $\textrm{Tl}$ from bulk amounts of $\textrm{HgO}$ target material between 550 and ${670}\,^\circ$°C. The separation efficiency was verified by $\gamma$-spectroscopy *via* the main $\gamma$-emissions of ${^{202}\textrm{Tl}}$ and co-produced ${^{203}\textrm{Hg}}$. This method generally provides a fast and reliable preparation of carrier-free, neutron-deficient $\textrm{Tl}$ radioisotopes (e.g., medically relevant ${^{201}\textrm{Tl}}$) from a proton-irradiated ${^\textrm{nat}\textrm{HgO}}$ matrix.

## Introduction

The production and chemical separation of carrier-free radionuclides is important in many scientific fields spanning from medicinal research to fundamental science. Typically, produced, carrier-free radionuclides are separated from bulk amounts of target material *via* various liquid-phase separation techniques [20]. Less commonly applied are gas-phase approaches utilizing the different gas-adsorption behavior of the desired carrier-free radionuclide and the constituents of the target material [35, 37]. One key advantage of gas-phase separations is the ability to separate different co-produced radionuclides within one separation [35].

Various chemical forms of $\textrm{Hg}$ have been previously irradiated with protons, namely, metallic $\textrm{Hg}$ [5, 9–12], $\textrm{HgS}$ [14], $\textrm{HgO}$ [4, 5, 7], and ${\textrm{Hg}_2\textrm{Cl}_2}$ [8]. Of these studies, most separated $\textrm{Hg}$ from $\textrm{Tl}$ *via* a combination of precipitation, liquid-liquid extraction, and/or ion-exchange chromatography [2, 4, 5, 8, 10–12]. Meanwhile, only Eichler and Domanov [9] utilized the volatile nature of metallic $\textrm{Hg}$ and successfully separated 92% of initially produced $\textrm{Tl}$ radioisotopes at ${450}\,^\circ$C under a ${\textrm{H}_2}$ stream [9]. However, the irradiation of metallic $\textrm{Hg}$ is prohibited at most proton accelerator facilities due to safety concerns regarding the toxicity and the comparably high volatility of $\textrm{Hg}$ already at room temperature. These concerns do not apply, however, to $\textrm{HgO}$, which makes it a promising target material for the production of $\textrm{Tl}$ radioisotopes by means of proton-irradiation.

The decomposition of $\textrm{HgO}$ to elemental $\textrm{Hg}$ and ${\textrm{O}_2}$ has been known for centuries, with its decomposition having led to the discovery of the element oxygen. Over the last century, the route, limitations, and kinetics of the $\textrm{HgO}$ decomposition have been thoroughly investigated [22, 26, 28, 29]. In macroscopic amounts, $\textrm{HgO}$ can rearrange at lower temperatures (${280}\,^\circ$C to ${380}\,^\circ$C) to ${\textrm{Hg}_2\textrm{O}}$ before decomposing [22] or it may directly decompose to elemental $\textrm{Hg}$ and ${\textrm{O}_2}$ at higher temperatures ($>{470}\,^\circ$C) [26]. The main decomposition temperature depends on the morphology, with red $\textrm{HgO}$ ($\approx {2}\,{\mu\textrm{m}}$ particles in ${20}\,{\mu \textrm{m}}$ aggregates) mainly decomposing at ${470}\,^\circ$C whereas yellow $\textrm{HgO}$ ($\approx {1}\,{\mu \textrm{m}}$ particles in ${5}\,{\mu \textrm{m}}$ aggregates) predominantly decomposes at ${280}\,^\circ$C [22, 26]. Notably, a small amount of red and yellow $\textrm{HgO}$ can decompose at both respective temperatures.

Presented here is the production and quantitative separation of carrier-free ${^{202}\textrm{Tl}}$ ($t_{1/2}={12.31(8)}\,\textrm{d}~$ [34]) from bulk amounts of proton-irradiated ${^\textrm{nat}\textrm{HgO}}$. ${^{202}\textrm{Tl}}$ was targeted for this proof of concept given its moderately long half-life and thus, the possibility of longer experimental times. In addition, co-produced ${^{203}\textrm{Hg}}$ ($t_{1/2}={46.610(10)}\,\textrm{d}~$ [17]) was used to trace the separation efficiency of the aimed at radioisotope of $\textrm{Tl}$ from the $\textrm{HgO}$ target material by $\gamma$-spectrometry. The presented approach can be easily adapted to other neutron-deficient radioisotopes of $\textrm{Tl}$, such as medically relevant ${^{201}\textrm{Tl}}$. Carrier-free quantities of this radioisotope are sought after as a diagnostic radionuclide for cardiovascular stress tests *via* SPECT imaging (Single Photon Emission Computed Tomography) [1, 16, 31]. Thus far, ${^{201}\textrm{Tl}}$ has been predominantly separated and purified for medical use from proton-irradiated $\textrm{Hg}$, $\textrm{Tl}$, $\textrm{Pb}$, and $\textrm{Bi}$, either directly or *via* longer-lived precursors [3, 5, 6, 21, 33, 35]. The popular indirect production route of ${^{201}\textrm{Tl}}$ ($t_{1/2}={3.0420(16)}\,\textrm{d}$ [18]) *via* ${^{201}\textrm{Pb}}$ ($t_{1/2}={9.33(5)}\,\textrm{h}$ [18]) as an intermediate from proton-irradiated $\textrm{Tl}$ (natural thallium or enriched in ${^{203}\textrm{Tl}}$) requires a substantial amount of time due to (1) the multi-step aqueous separation of $\textrm{Pb}$ from the bulk $\textrm{Tl}$ target material, (2) a ${32}\,\textrm{h}$ waiting period for most of the ${^{201}\textrm{Pb}}$ having decayed to ${^{201}\textrm{Tl}}$, and (3) the separation of in-grown ${^{201}\textrm{Tl}}$ from the remaining $\textrm{Pb}$ radioisotopes [2, 3, 24, 33]. With the separation procedure presented herein, the preparation time of carrier-free ${^{201}\textrm{Tl}}$ can be reduced significantly.

Furthermore, ${^{202}\textrm{Tl}}$ can be readily used for homolog studies prior to experiments aimed at the chemical characterization of superheavy elements ($Z\ge 104$) as the newest members of the periodic table [30]. In particular, carrier-free radioisotopes of $\textrm{Tl}$, i.e., the lighter homolog of nihonium (Nh, $Z=113$), are currently needed for preliminary studies in preparation of a later envisaged experiment with $\textrm{Nh}$ [27].

## Materials and methods

### Target preparation

Three targets were prepared by pressing ${151(1)}\,\textrm{mg}$ ${^\textrm{nat}\textrm{HgO}}$ powder (*ThermoFisher Scientific*, +98.5% red) into ${6}\,\textrm{mm}$ disks with a thickness of ${0.5}\,\textrm{mm}$. During proton irradiation at the IP2 beamline of the High-Intensity Proton Accelerator facility at the Paul Scherrer Institute (PSI) [13], the target material reaches elevated temperatures as a consequence of the energy loss of the intense proton beam inside the target. Since $\textrm{HgO}$ may decompose to elemental $\textrm{Hg}$ and ${\textrm{O}_2}$ starting from ${280}\,^\circ$C [22, 26], the $\textrm{Al}$ target holder was anodized prior to irradiation. This created a comparably thick insulating ${\textrm{Al}_2\textrm{O}_3}$ passivation layer (${10}\,{\mu\textrm{m}}~\textrm{to}~{15}\,{\mu\textrm{m}}$) acting as a safety precaution against the amalgamation between elemental $\textrm{Al}$ and potentially evolving metallic $\textrm{Hg}$.

### Proton beam irradiation

**Fig. 1:**
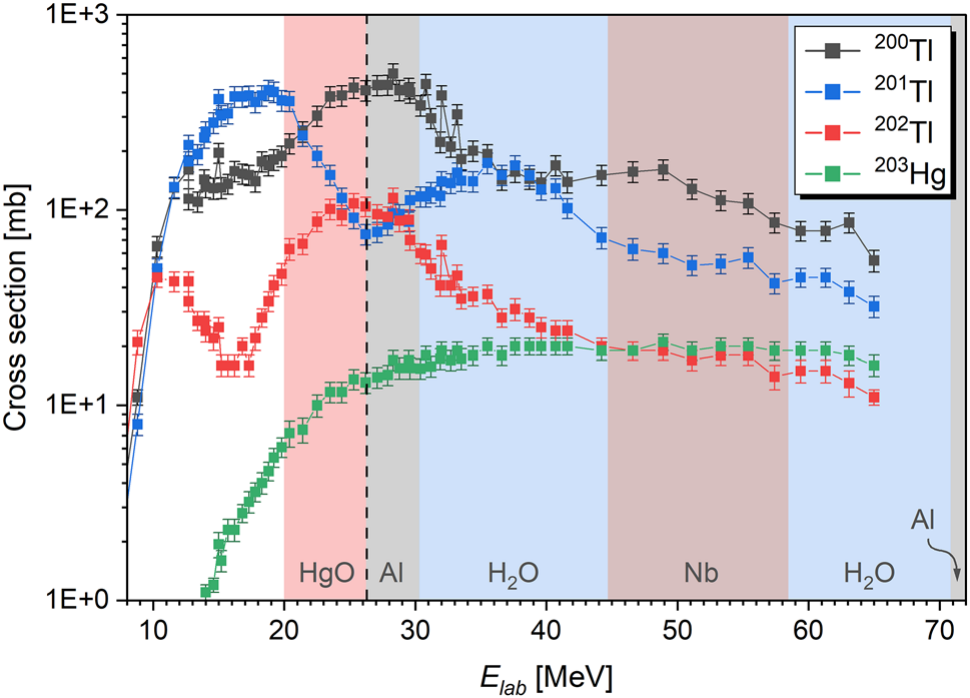
Cumulative excitation functions as functions of the proton beam energy (laboratory frame) for ${^{200}\textrm{Tl}}$ (black squares), ${^{201}\textrm{Tl}}$ (blue circles), ${^{202}\textrm{Tl}}$ (red triangles), and ${^{203}\textrm{Hg}}$ (green diamonds) [14] as produced in the proton-induced reactions ${^\textrm{nat}\textrm{Hg}}$(p,*x*n)${^{200,201,202}\textrm{Tl}}$ (with $x=1-5$) and ${^{204}\textrm{Hg}}$(p,pn)${^{203}\textrm{Hg}}$. The chosen proton beam energy on target of $\approx {26}\,\textrm{MeV}$ (dashed black line) is indicated, as well as individual energy losses in different degrading layers prior, starting from the initial proton beam energy of ${72}\,\textrm{MeV}$; the degrading layers (from right to left) are a ${0.6}\,\textrm{mm}$ $\textrm{Al}$ vacuum window (gray shaded area), a ${12}\,\textrm{mm}$ ${\textrm{H}_2\textrm{O}}$ cooling water gap (blue shaded area), the ${2.2}\,\textrm{mm}$ $\textrm{Nb}$ degrader (brown shaded area), another ${9}\,\textrm{mm}$ $\textrm{H}_2\textrm{O}$ cooling water gap (blue shaded area), the ${1.0}\,\textrm{mm}$ thick $\textrm{Al}$ target holder cap (gray shaded area), and the ${0.5}\,\textrm{mm}$ thick $\textrm{HgO}$ target (red shaded area) [13] (all energy losses were calculated with SRIM [36]).

Herein, ${^{202}\textrm{Tl}}$ ($t_{1/2}={12.4706(55)}\,\textrm{d}~$ [34]) was targeted with alternative reaction channels leading to the co-production of ${^{203}\textrm{Hg}}$ ($t_{1/2}={46.610(10)}\,\textrm{d}~$ [17]). These two radionuclides were produced predominantly in the proton-induced nuclear reactions ${^{204}\textrm{Hg}}$(p,3n)${^{202}\textrm{Tl}}$ and ${^{204}\textrm{Hg}}$(p,pn)${^{203}\textrm{Hg}}$ [14] (see Fig. 1). To maximize the ${^{202}\textrm{Tl}}$ production yield, a $\approx {26}\,\textrm{MeV}$ proton beam energy was chosen [14]. This was achieved by reducing the primary proton beam energy of ${72}\,\textrm{MeV}$ at IP2 using a ${2.2}\,\textrm{mm}$ $\textrm{Nb}$ degrader [13] in addition to the permanent ${0.6}\,\textrm{mm}$ thick $\textrm{Al}$ vacuum window and the $12+{9}\,\textrm{mm}$ cooling water gaps prior to the ${1.0}\,\textrm{mm}$ $\textrm{Al}$ cap of the target capsule (see shaded areas in Fig. 1, indicating the individual energy losses of the proton beam in each layer). Each target was irradiated by $\approx {3.75}\times 10^{16}\,\textrm{protons}$ with an average proton beam intensity of ${20}\,{\mu \textrm{A}}$. Produced activities ranged from ${324}\,\textrm{kBq}$ to ${474}\,\textrm{kBq}$. After the end-of-bombardment (EOB), the targets were stored for at least two weeks prior to the separation studies. This led to the decay of short-lived nuclear reaction products, leaving only relatively long-lived produced radionuclides (see Table 1).

**Table 1:** Remaining radionuclides (RN) and their main $\gamma$-lines as produced in proton-induced nuclear reactions (NR) after a storage time of two weeks, where $x=1-5$ indicates the number of evaporated neutrons and *y* highlights multi-particle emissions (e.g., ${^{198}\textrm{Hg}}$(p,2pn)${^{196}\textrm{Au}}$); associated references are stated directly in the Table.

RN	$t_{1/2}$	Main $\gamma$-line (keV)	$I_{\gamma }$ (%)	NR	Reference
${^{196}\textrm{Au}}$	${6.1669(6)}\,\textrm{d}$	355.73(5)	87(3)	${^\textrm{nat}\textrm{Hg}}$(p,*y*)	[32]
${^{198}\textrm{Au}}$	${2.6941(2)}\,\textrm{d}$	411.80205(17)	95.62(6)	${^\textrm{nat}\textrm{Hg}}$(p,*y*)	[15]
${^{200}\textrm{Tl}}$	${26.1(1)}\,\textrm{h}$	367.942(10)	88.49(22)	${^\textrm{nat}\textrm{Hg}}$(p,*x*n)	[19]
${^{201}\textrm{Tl}}$	${3.0420(16)}\,\textrm{d}$	167.43(7)	10.00(10)	${^\textrm{nat}\textrm{Hg}}$(p,*x*n)	[18]
${^{202}\textrm{Tl}}$	${12.4706(55)}\,\textrm{d}$	439.56(1)	91.5(10)	${^\textrm{nat}\textrm{Hg}}$(p,*x*n)	[34]
${^{203}\textrm{Hg}}$	${46.610(10)}\,\textrm{d}$	279.1952(10)	81.56(6)	${^{204}\textrm{Hg}}$(p,pn)	[17]

### Experimental setup and methods

**Fig. 2:**
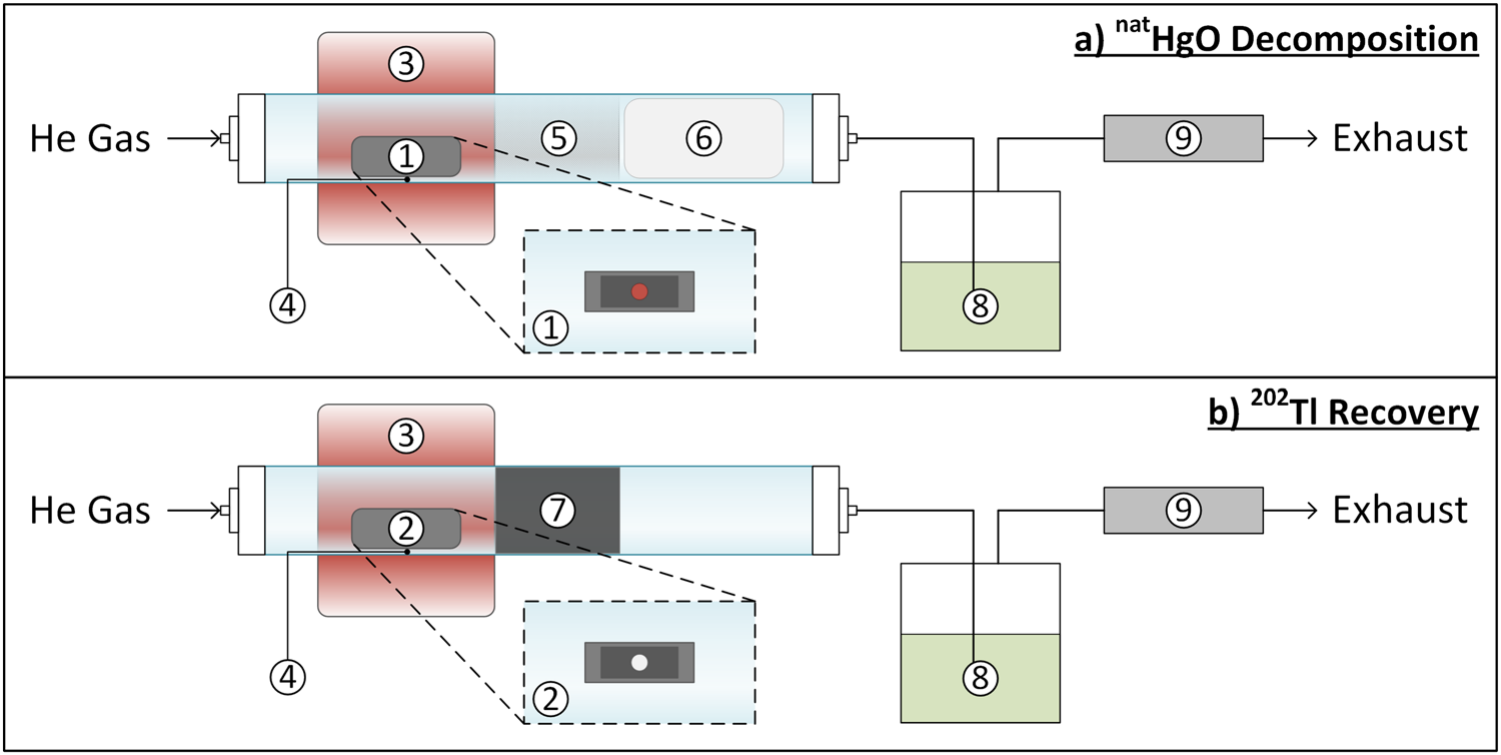
Schematic of the setups for a) ${^\textrm{nat}\textrm{HgO}}$ decomposition and b) ${^{202}\textrm{Tl}}$ recovery. The $\textrm{He}$ carrier gas flow was controlled by a mass flow controller set to ${50}\,\textrm{mL}/\textrm{min}$ before entering the fused silica column with either (1) the $\textrm{Ta}$ crucible containing the irradiated ${^\textrm{nat}\textrm{HgO}}$ target or (2) the $\textrm{Ta}$ crucible containing the remaining ${^{202}\textrm{Tl}}$ after the concluded ${^\textrm{nat}\textrm{HgO}}$ decomposition, which was heated by (3) a custom-made oven with (4) a type K thermocouple (between fused silica column and oven); beyond the oven, (5) metallic $\textrm{Hg}$ condensed on the bare fused silica surface (i.e., during decomposition, setup a) or (7) ${^{202}\textrm{Tl}}$ adsorbed on $\textrm{Ta}$ foils (i.e., during ${^{202}\textrm{Tl}}$ recovery, setup b). Safety features to prevent the escape of $\textrm{Hg}$ or any other volatile radionuclide included (6) a quartz wool trap, (8) a $\textrm{Ag}$-coated zeolite trap, and (9) a charcoal trap.

**Fig. 3:**
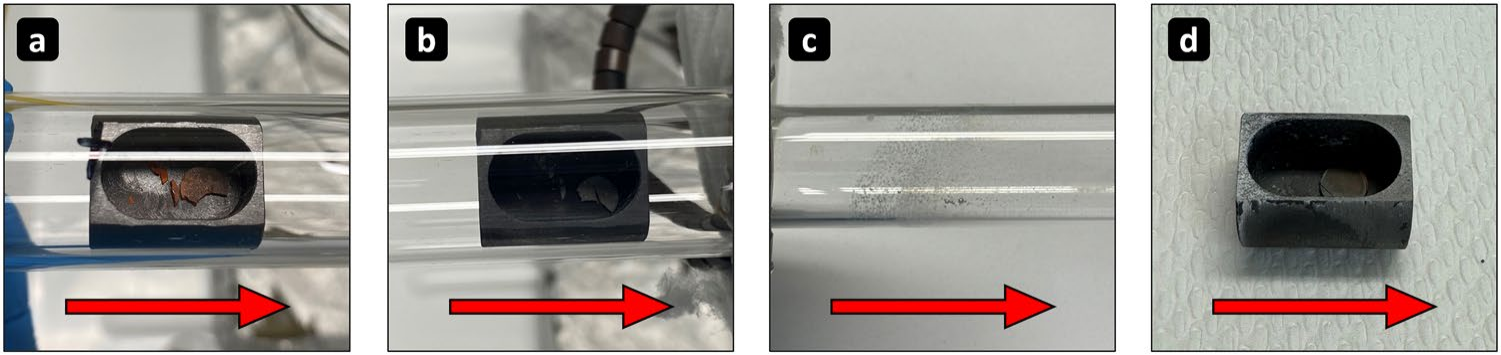
Pictures taken of (**a**) an irradiated, pressed red ${^\textrm{nat}\textrm{HgO}}$ target placed within the $\textrm{Ta}$ crucible prior to the thermal decomposition of ${^\textrm{nat}\textrm{HgO}}$ at $>{500}\,^\circ$C, (**b**) the $\textrm{Ta}$ crucible after decomposition, (**c**) the elemental $\textrm{Hg}$ condensed as droplets within the fused silica column after decomposition, and (**d**) the $\textrm{Ta}$ crucible after the transfer of remaining ${^{202}\textrm{Tl}}$ at $>{750}\,^\circ$C (note the white oxidized $\textrm{Ta}$ surface on the upstream side relative to the flow direction of the carrier gas). The flow direction of the carrier gas is indicated with red arrows.

All experiments were conducted with a custom-made $\textrm{Ta}$ crucible machined from a $\textrm{Ta}$ rod (${16}\,\textrm{mm}$ diameter, ${20}\,\textrm{mm}$ length) longitudinally cut and hollowed (${1}\,\textrm{mL}$ crucible volume; see Figs. 2 and 3) to fit within the fused silica column (${20}\,\textrm{mm}$ inner diameter). Prior to their first use, the $\textrm{Ta}$ crucible and $\textrm{Ta}$ foils (i.e., used for the ${^{202}\,\textrm{Tl}}$ recovery) were cleaned with ethanol and thermally treated at $>{200}\,^\circ$C under a $\textrm{He}$ carrier gas flow of ${50}\,\textrm{ml}/\textrm{min}$ ($\textrm{He}$ 5.0) to remove any remaining organic material (e.g., from machining). The $\textrm{Ta}$ crucible containing the irradiated target was aligned to a custom-built oven (KANTHAL-based; ${25}\,\textrm{mm}$ opening, ${50}\,\textrm{mm}$ length, $\approx {1100}\,^\circ$C maximum operating temperature). The temperature was constantly monitored by a type K thermocouple placed between the outer wall of the fused silica column and the oven, aligned with the $\textrm{Ta}$ crucible inside.

The decomposition of ${^\textrm{nat}\textrm{HgO}}$ as well as the recovery of ${^{202}\textrm{Tl}}$ from the $\textrm{Ta}$ crucible were both conducted under a $\textrm{He}$ carrier gas flow of ${50}\,\textrm{mL}/\textrm{min}$ ($\textrm{He}$ 5.0). During the decomposition step (see Fig. 2a), once the oven had reached ${500}\,^\circ$C after $\approx {20}\,\textrm{min}$, the $\textrm{Hg}$ started to condense in the form of small droplets in the downstream direction on the inner wall of the fused silica column (see Fig. 3c). To prevent any $\textrm{Hg}$ from escaping following the thermal decomposition, a large quartz wool plug was placed downstream of the oven at the end of the fused silica column. The carrier gas then passed through an $\textrm{Ag}$-coated zeolite trap and an additional charcoal-based filter unit (see Fig. 2). The oven was maintained at $>{500}\,^\circ$C for at least ${15}\,\textrm{min}$ to ensure full thermal decomposition of $\textrm{HgO}$. The apparatus was subsequently cooled under the continued $\textrm{He}$ carrier gas flow until the $\textrm{Ta}$ crucible had reached room temperature (after $\approx {1}\,\textrm{h}$). Then, the carrier gas flow was stopped and the $\textrm{Ta}$ crucible removed from the upstream direction to avoid contact with the condensed $\textrm{Hg}$ phase downstream. Following the extraction of the $\textrm{Ta}$ crucible, the fused silica column was unmounted and all metallic $\textrm{Hg}$ droplets were combined and collected by turning the fused silica column along its axis until only one large $\textrm{Hg}$ droplet remained.

The quality of separation of the irradiated ${^\textrm{nat}\textrm{HgO}}$ target material from the $\textrm{Tl}$ radioisotopes produced therein was traced *via* the main $\gamma$-lines of ${^{202}\textrm{Tl}}$ and ${^{203}\textrm{Hg}}$ (see Table 1). High-purity $\textrm{Ge}$ $\gamma$-detectors (i.e., either a BE2825 from *CANBERRA* or a EGC 35-200-R from *Eurisys Mesures*) were employed for all $\gamma$-spectrometric measurements. The $\gamma$-spectra of the irradiated target material in a plastic vial, as well as within the $\textrm{Ta}$ crucible before the thermal decomposition, were taken as reference $\gamma$-spectra for the separated $\textrm{Hg}$ and the recovered $\textrm{Tl}$ respectively. This allowed for a direct comparison to the recovered $\textrm{Tl}$ and $\textrm{Hg}$ fractions following the $\textrm{HgO}$ decomposition (i.e., to the separated ${^{202}\textrm{Tl}}$ in the $\textrm{Ta}$ crucible and to the collected metallic $\textrm{Hg}$ in an identical plastic vial as used before). Identical measurement geometries ensured comparable detection efficiencies.

The recovery of ${^{202}\textrm{Tl}}$ (see Fig. 2b) from the $\textrm{Ta}$ crucible made use of the same setup, but within a separate fused silica column. With the quartz wool plug removed, $\textrm{Ta}$ foils lined (spanning $\approx {10}\,\textrm{cm}$) the inside of the fused silica column just beyond the oven in the downstream direction of the carrier gas to collect transported ${^{202}\textrm{Tl}}$. To desorb the ${^{202}\textrm{Tl}}$ from the $\textrm{Ta}$ crucible, the oven was heated to $>{725}\,^\circ$C for ${1}\,\textrm{h}$ to ${2}\,\textrm{h}$ under a ${50}\,\textrm{mL}/\textrm{min}$ $\textrm{He}$ carrier gas flow ($\textrm{He}$ 5.0). After the apparatus had cooled down to room temperature, the remaining activities in the $\textrm{Ta}$ crucible and those transferred onto the foils were measured again by $\gamma$-spectrometry. In order to probe ideal experimental conditions for the ${^{202}\textrm{Tl}}$ recovery following the $\textrm{HgO}$ decomposition, a number of temperatures and experimental times were tested to track the ${^{202}\textrm{Tl}}$ removal from the $\textrm{Ta}$ crucible (see Table 2).

**Table 2:** The recovery of ${^{202}\textrm{Tl}}$ from the $\textrm{Ta}$ crucible as a function of the applied oven temperature under a ${50}\,\textrm{mL}/\textrm{min}$ $\textrm{He}$ carrier gas flow relative to the initial $\gamma$-spectrometric measurement of the $\textrm{Ta}$ crucible with the proton-irradiated ${^\textrm{nat}\textrm{HgO}}$ target inside (i.e., recovery calculated as the difference between initial and remaining activity inside the crucible); experiments marked with an asterisk ($^{*}$) correspond to actual decomposition runs (see Fig. 2a); all remaining entries represent recovery experiments (see Fig. 2b).

Target #	Separation	Temperature ($^\circ$C)	Duration (h)	Recovery (%)
1 $^{*}$	1	550	0.8	0
1	2	725	1.75	7
1	3	770	1	53
1	4	850	2	84
2 $^{*}$	1	670	2	13
2	2	780	1.8	43
3 $^{*}$	1	600	0.3	14
3	2	850	0.75	48

## Results and discussion

**Fig. 4:**
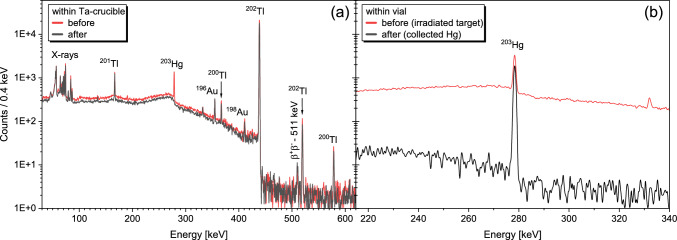
Exemplary $\gamma$-spectra of irradiated ${^\textrm{nat}\textrm{HgO}}$ target #3 (see also Table 2) within a plastic vial (red line, b) and within the $\textrm{Ta}$ crucible (red line, a) prior to the heat treatment in the course of the ${^\textrm{nat}\textrm{HgO}}$ decomposition as well as afterwards of the $\textrm{Ta}$ crucible (black line, a) and the collected metallic $\textrm{Hg}$ fraction in an identical plastic vial (black line, b). The proton-induced nuclear reaction products as well as the X-rays of $\textrm{Ta}$, $\textrm{Hg}$, and $\textrm{Pb}$ detected are indicated accordingly [15, 17–19, 32, 34].

The quantitative separation of $\textrm{Hg}$ (traced by co-produced ${^{203}\textrm{Hg}}$) from carrier-free ${^{202}\textrm{Tl}}$ was successful (see Fig. 4a). This was possible due to the sufficiently large difference between the decomposition temperature of $\textrm{HgO}$ [22, 26, 28] and the temperature needed for $\textrm{Tl}$ to desorb from the $\textrm{Ta}$ surface [23, 25]. Only when the temperature during the $\textrm{HgO}$ decomposition reached $\ge {600}\,^\circ$C were there small amounts of ${^{202}\textrm{Tl}}$ transferred out of the $\textrm{Ta}$ crucible (see Table 2).

Compared to the initial activity of the irradiated ${^\textrm{nat}\textrm{HgO}}$ target #3, only $\approx 65\%$ of ${^{203}\textrm{Hg}}$ could be recovered from the fused silica column after the $\textrm{HgO}$ decomposition (see Fig. 4b). The remaining $\textrm{Hg}$ was trapped within the quartz wool plug (as verified by $\gamma$-spectroscopic measurements of the quartz wool plug, the fused silica column, the $\textrm{Ag}$-coated zeolite trap, and the charcoal trap) and could not be collected by simply rolling the fused silica column along its axis. Similar losses were encountered with all other targets.

A maximum of $\approx 84\%$ of ${^{202}\textrm{Tl}}$ could be recovered from the $\textrm{Ta}$ crucible at ${850}\,^\circ$C and sufficiently long heating times (at least ${2}\,{\textrm{h}}$, see Table 2). The ${^{202}\textrm{Tl}}$ deposited on $\textrm{Ta}$ foils placed in the downstream direction. Given the reduction capability of a hot $\textrm{Ta}$ surface at $\ge {800}\,^\circ$C, it is expected that $\textrm{Tl}$ desorbs from the hot surface of the $\textrm{Ta}$ crucible in its elemental state. The small loss of ${^{202}\textrm{Tl}}$ can be attributed to multiple factors such as the relatively high walls of the $\textrm{Ta}$ crucible and the thereof resulting inefficient flushing of the inner volume by the carrier gas under laminar flow conditions. The latter can be easily improved by a re-design of the $\textrm{Ta}$ crucible with lower walls on either side. Since elemental $\textrm{Tl}$ features comparably high adsorption enthalpies on all metal surfaces [9], a variety of metals may be used as crucible material or as the final deposition material. This allows for an easy and direct customization of the herein presented approach.

With regard to medical applications, radioisotopes of $\textrm{Tl}$ are needed as $\textrm{TlCl}$ in saline solutions ($0.9\%$ $\textrm{NaCl}$ solution) [3, 12]. To extract carrier-free radioisotopes of $\textrm{Tl}$ from a $\textrm{Ta}$ surface, the procedure by Goetz et al. [12] can be employed. Therein, the authors irradiated metallic $\textrm{Hg}$ within a $\textrm{Ta}$ container with protons; after pouring out the metallic $\textrm{Hg}$, the $\textrm{Tl}$ radioisotopes were extracted by washing the $\textrm{Ta}$ container multiple times with ${1}\,{\textrm{mL}}$ of ${3}\,{\textrm{M}}$ $\textrm{HCl}$ [12].

## Conclusion

Carrier-free ${^{202}\textrm{Tl}}$ was quantitatively separated from proton-irradiated ${^\textrm{nat}\textrm{HgO}}$ target material (radionuclidically pure, with ${^{203}\textrm{Hg}}$ being below the detection limit using $\gamma$-spectrometry). This was achieved leveraging the temperature difference required for the thermal decomposition of the $\textrm{HgO}$ target material and the desorption of elemental $\textrm{Tl}$ from a $\textrm{Ta}$ surface. The separated ${^{202}\textrm{Tl}}$ was recovered from the $\textrm{Ta}$ crucible by isothermally heating beyond ${725}\,^\circ$C. This approach enables a straight-forward provision of carrier-free radioisotopes of $\textrm{Tl}$, e.g., for chemical studies with $\textrm{Tl}$ as the lighter homolog of superheavy element $\textrm{Nh}$. Furthermore, the presented gas-phase separation can be easily adopted to medically relevant ${^{201}\textrm{Tl}}$, which would reduce the preparation time considerably when compared to currently employed production and separation routes.

## Data Availability

The data supporting this study’s findings are available from the corresponding author upon request.
